# Assessment of Antibiotic Levels, Multi-Drug Resistant Bacteria and Genetic Biomarkers in the Waters of the Rio Grande River Between the United States-Mexico Border

**DOI:** 10.5696/2156-9614-9.23.190912

**Published:** 2019-08-22

**Authors:** Maria D. Fuentes, Stephanie Gutierrez, Daniella Sahagun, Jose Gomez, Jose Mendoza, Cameron C. Ellis, Stephanie Bauer, Jonathan Blattner, Wen-Yee Lee, Maria Alvarez, Delfina C. Domínguez

**Affiliations:** 1 Interdisciplinary Health Sciences PhD Program, The University of Texas at El Paso, El Paso, Texas; 2 Biology Department, El Paso Community College, El Paso, Texas; 3 Department of Chemistry, The University of Texas at El Paso, El Paso, Texas; 4 Department of Clinical Laboratory Sciences, Department of Public Health Sciences, The University of Texas at El Paso, El Paso, Texas

**Keywords:** antimicrobial resistance, antibiotics, extended-spectrum beta-lactamase (ESBL), Rio Grande River, integrons, mobile genetic elements, horizontal gene transfer

## Abstract

**Background.:**

The worldwide emergence of multi-drug resistant bacteria has become a health crisis, as fewer or sometimes no antimicrobial agents are effective against these bacteria. The Rio Grande River is the natural boundary between the United States (US) and Mexico. It spans a border region between Texas, New Mexico and Mexico. Underserved populations on the Mexican side use the river for recreational purposes, while on the US side, the river is used for irrigation and as a source of drinking water.

**Objectives.:**

The purpose of the present study was to evaluate the concentration of antibiotic residues, to determine the presence of genetic elements conferring antibiotic resistance and to characterize multi-drug resistant bacteria in the waters of the Rio Grande River.

**Methods.:**

Water samples were obtained from the Rio Grande River. Deoxyribonucleic acid (DNA) was extracted from both isolated bacteria and directly from the water. Amplification of selected genetic elements was accomplished by polymerase chain reaction. Identification and isolation of bacteria was performed through MicroScan autoSCAN-4. Fecal contamination was assessed by IDEXX Colilert. Antibiotic residues were determined by liquid chromatography and mass spectrometry.

**Results.:**

Antibiotics were found in 92% of both water and sediment samples. Antibiotic concentrations ranged from 0.38 ng/L - 742.73 ng/L and 0.39 ng/l - 66.3 ng/g dry weight in water and sediment samples, respectively. Genetic elements conferring resistance were recovered from all collection sites. Of the isolated bacteria, 91 (64.08%) were resistant to at least two synergistic antibiotic combinations and 11 (14.79%) were found to be resistant to 20 or more individual antibiotics. Fecal contamination was higher during the months of April and July.

**Conclusions.:**

The 26 km segment of the Rio Grande River from Sunland Park NM to El Paso, TX and Juarez, Mexico is an area of concern due to poor water quality. The presence of multidrug resistant bacteria, antibiotics and mobile genetic elements may be a health hazard for the surrounding populations of this binational border region. Policies need to be developed for the appropriate management of the environmental natural resources in this border region.

**Competing Interests.:**

The authors declare no competing financial interests.

## Introduction

Antimicrobial resistance (AR) represents one of the most important global challenges in public health. It has been estimated that by the year 2050, deaths by complications related to infectious diseases will increase to as many as 10 million per year.^1^ The microorganisms currently reported to represent major health threats due to increased AR, not only in the US but around the world, include carbapenem resistant enterobacteria, methicillin-resistant Staphylococcus aureus, and extended spectrum β-lactamase (ESBL) producing bacteria.^2^,^3^ These multidrug resistant (MDR) bacteria are disseminating rapidly through mobile genetic elements not only in health care, but in the environment worldwide.

Extended spectrum β-lactamases are plasmid-mediated enzymes produced by gram-negative bacteria that render β-lactam antibiotics (e.g. penicillins, cephalosporins, carbapenems) inactive.^4^ Furthermore, these enzymes exhibit co-resistance to many other classes of antibiotics, limiting therapeutic options. Infections caused by ESBLs range from urinary tract infections to life threatening sepsis.^5^ Consequences of AR infections in humans include longer hospitalization, intake of more toxic and expensive medications resulting in higher medical costs and higher mortality rates, and patient complications, such as spreading of infection to different body sites. Due to technical issues, ESBL detection represents a challenge.^6^ However, a combination of culture-based methods and molecular technology offers the best detection. Among the most commonly antimicrobial resistant genes (ARG) encoding ESBL enzymes include TEM, SHV, and CTX-M.^7^ The most frequently isolated ESBLresistant bacteria are Escherichia coli (E. coli), Klebsiella pneumoniae *and* Pseudomonas aeruginosa.^7^

While there are numerous reports on AR research in health care settings, limited work has been performed examining the environment as a major reservoir of antibiotic resistant bacteria and the impact on public health. With the goal of understanding the extent of AR in the ecosystem, the present study aimed to first identify multidrug resistant organisms, ARG and the most common antimicrobial residues found in waters of the Rio Grande River, which is the main source of potable water for a population of nearly 3 million people in the United States-Mexico border region.

### Driving factors of resistance in the environment

Although AR has been largely attributed to the overuse and misuse of antibiotics, there are many other factors involved in the antibiotic resistance crisis that contribute to the development and dissemination of resistant genes in the environment.^3^,^8^–^10^ Some of these include the use of antibiotics in agriculture, aquaculture and in cattle as prophylaxis and growth promoters for commercial purposes.^3^,^11^ Animals can excrete between 50% and 100% of the administered dose of some antimicrobial compounds within several days of treatment.^12^ The resulting agricultural pollution can easily reach humans via fish/meat consumption. Surface water runoff and other natural physical forces can disseminate the compounds in animal feces throughout the environment.^10^ Human antibiotic intake can also enter surface waters directly from the effluents of wastewater treatment plants (WWTPs) by excretion, flushing of old prescriptions and medical waste from clinics and hospitals.^12^ Hence, surface water is now identified as an important reservoir of AR bacteria contributing to the evolution and further spread of resistant organisms.^13^–^15^

**Abbreviations**
*AR*: Antibiotic resistance
*ARG*: Antimicrobial resistant genes
*DNA*: Deoxyribonucleic acid
*ESBL*: Extended spectrum β-lactamase
*MDR*: Multidrug resistance
*MPN*: Most probable number
*m/z*: Mass over charge
*TCEQ*: Texas Commission of Environmental Quality
*WWTP*: Wastewater treatment plants

Other anthropogenic activities that contribute to the spread of AR bacteria include urbanization, worldwide travel, WWTPs and industrial effluents contaminated with pharmaceutical products and heavy metals.^10^,^14^,^16^,^17^ Genetic factors contributing to the rapid dissemination of AR involve the ability of ARG and mobile genetic elements such as plasmids, transposons and integrons to transfer easily into other bacteria by a variety of genetic mechanisms.^4^,^12^,^13^,^18^ The transfer of genetic material between bacterial cells is induced by stressors such as antibiotics, biocides and heavy metals.^10^,^19^ Furthermore, reports indicate that sub-lethal concentrations of antibiotics and heavy metals found in polluted environments can contribute to bacterial resistance by recruitment of resistant genes carried in mobile elements, and by maintaining MDR plasmids in host bacteria and allowing them to survive.^19^–^21^

### United States-Mexico border region

The United States (US)-Mexico border encompasses 100 km north and south of the international boundary and is comprised of two sovereign nations, four states in the US and six states in Mexico.^22^ Along the border, 95% of the population lives in sister communities.^23^ It is estimated that about 29% of US border residents live below the poverty level.^24^ The Texas border region has high poverty rates, higher rates of uninsured and is medically underserved.^23^ The poverty rate in El Paso, Texas is estimated to be 23% and approximately 30% of residents are uninsured.^25^

The Rio Grande River is the natural boundary between the United States and Mexico. It spans the border region between Texas, New Mexico and Mexico. The Rio Grande River provides a major source of potable and agricultural water in the New Mexico, Texas and Mexico border region. Previous reports from one of our laboratories showed that the Rio Grande exceeds standards for fecal contamination and chemical toxicity including heavy metals.^26^,^27^ Factors that may contribute to the microbial burden and the release of antimicrobial agents and pharmaceuticals into the Rio Grande include cattle farming and ranching, horse racetracks, WWTPs, septic disposal systems, animal feeding operations and other activities related to urbanization along the river. The presence of antibiotic resistant bacteria in the Rio Grande River may lead to an increased number and severity of infections, frequency of treatment failure, allergies, and alteration of intestinal flora.

The presence of antimicrobial residues in the environment is a growing public health concern. Antibiotic residues can persist in the environment after direct disposal of unused or expired medication from pharmaceutical plants, hospitals and the community.^28^ It is estimated that up to 90% of an antibiotic is not completely metabolized in humans after ingestion and can be excreted through urine or feces into the environment via domestic sewage and WWTPs.^29^ These compounds can remain in the soil and water (both surface and groundwater) for long periods of time and appear to have the ability to generate new by-products under the new aquatic environment.^30^–^32^ The presence of these antibiotic residues and their metabolites may be a novel source for antibiotic resistance development in re-emergent pathogens favoring mutations, genetic recombination and horizontal gene transfer.^10^,^33^,^34^

To manage the antibiotic resistance crisis, it is important to identify and quantify antibiotic residues, AR bacteria and mobile genetic elements in the environment; to investigate the different mechanisms of bacterial spreading in the environment and their possible effects on human health.

## Methods

Sampling sites were selected according to urbanization and other anthropogenic activities that could play an important role in the dissemination of AR in the environment. This project was approved by the Institutional Biosafety Committee at the University of Texas at El Paso.

### Water sampling

**Figure 1:**
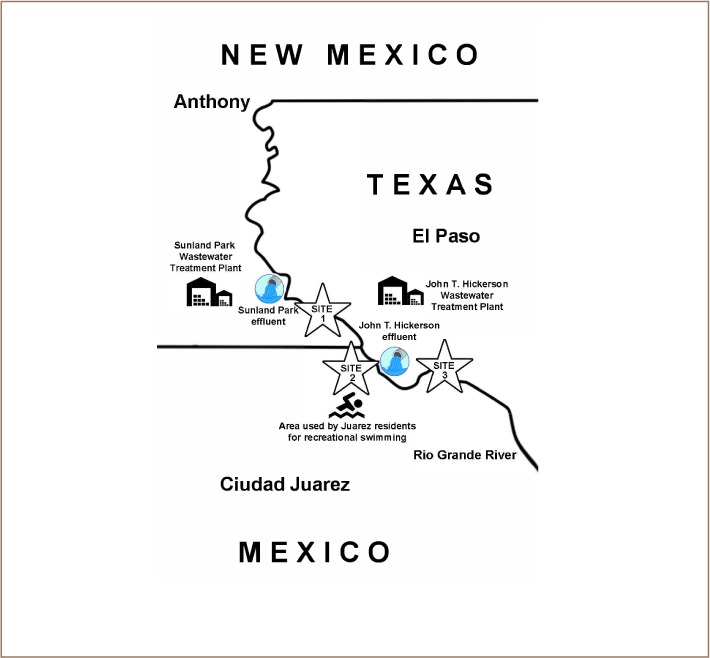
Rio Grande sampling sites: Site 1: Sunland Park area near waste water treatment plant (NM); Site 2: Riverbend/Courchesne area near waste water treatment plant effluent (Texas); Site 3: Anapra area (Juarez, Mexico and Texas)

Water samples were collected in one-quart cubitainers using the grab method during the months of February, April, July, September, and December 2017. Three river sites were sampled along a 26 km segment of the Rio Grande River between Sunland Park, NM and El Paso, TX. River areas selected were: Site 1) Sunland Park, NM area, upstream from the Sunland Park Drive Bridge and impacted by effluents from Sunland Park WWTP, which is responsible for the waste water system of Sunland Park and Santa Teresa, NM (coordinates: 31.798843, −106.557647); Site 2) Courchesne Bridge area, located 50 m downstream from Courchesne Bridge in El Paso, TX and 1 km downstream from the Montoya Drain, a canal in the county of El Paso where many dairy, cotton, cattle and farming products are processed (coordinates: 31.801885, −106.540540); and Site 3) River Bend area near Sunset Heights in El Paso, TX located on the US side of the river across from a public park located on the Mexican side, and selected for the high recreational activities of families and children on the river (Coordinates: 31.768277, −106.511933). Water samples were sent to laboratory facilities at the University of Texas at El Paso and El Paso Community College for molecular/chemical and microbiological analysis, respectively *(Figure 1)*.

### Assessment of fecal contamination

The most probable number (MPN) of E. coli/100 ml was determined using the IDEXX Colilert system (IDEXX Laboratories, Inc., Westbrook, Maine USA) as an indicator of fecal contamination according to manufacturer's instructions.

### Identification of bacterial isolates and antibiotic profiles

Collected water samples were filtered through 0.45 μm Millipore membrane filters. Filters were placed on selective and differential media including bile esculin azide agar, modified mTEC agar (Difco^™^), mEndo agar and mannitol salt agar and incubated for 24 hours at 37°C. Isolated colonies were re-streaked for isolation and gram stained. Once isolated, colonies were analyzed using the MicroScan autoSCAN-4 automated bacterial identification system. MicroScan panels NBPC 34 were used to identify gram negative isolates and PBPC 20 panels for gram positive isolates as well as for determination of their corresponding antimicrobial susceptibility patterns.

### Deoxyribonucleic acid extraction from AR bacterial isolates

Gram negative bacterial isolates showing resistance to two or more β-Lactam antibiotics such as penicillins, broad spectrum cephalosporins and carbapenems were selected for further molecular characterization of ESBL genes (TEM, CTX-M and SHV). Resistant bacteria were cultured in Luria-Bertani broth and incubated at 35°C for 24 hours. Deoxyribonucleic acid (DNA) extraction was performed by suspending a colony from an overnight grown culture on trypticase soy agar, in 50 μl water and boiling at 100°C for 10 minutes.^35^ The integrity of the genomic DNA was assessed by electrophoresis in a 1.5% agarose gel in Tris-acetate-EDTA buffer.

### Direct DNA extraction from water samples

A sample of 1500 ml of water was collected each month. Water samples were filtered through a 0.45 μm Millipore filter using a vacuum filtration unit. Deoxyribonucleic acid extraction was performed using the Rapid Water DNA Isolation Kit (MoBio Laboratories, Solana Beach, CA, USA) following the manufacturer's instructions.

### Identification of resistance genes and mobile genetic elements by polymerase chain reaction

Multiplex polymerase chain reaction amplification was used to investigate the presence of the most common genes encoding ESBLs, TEM, SHV and CTX-M. Polymerase chain reaction amplification was performed following the protocol of Monstein *et al*. with slight modifications.^36^ Polymerase chain reaction amplification conditions were as follows: initial denaturation at 95°C for 15 minutes and 30 cycles at 95°C for 30 seconds, annealing at 55°C for 30 seconds, extension at 72°C for 2 minutes, followed by a final extension at 72°C for 5 minutes. Deoxyribonucleic acid from control ATCC organisms (CTX BAA-2326, SHV BAA199 and TEM BAA-196) were included in each run. Polymerase chain reaction samples were run in 1.5% agarose gel Trisacetate-ethylenediaminetetraacetic acid (EDTA) buffer for 90 minutes at 75 volts. Polymerase chain reaction for Class 1 and 2 integrons were performed following Kotlarska's protocol.^28^

Controls for integrons were included in each run (Int-1 E. clocae E705 and Int-2 A. baumannii A98). Carbapenem-producing isolates were screened using BBL^™^ CHROMagar^™^ KPC, which is a chromogenic medium designed to detect reduced sensitivity to carbapenem agents. Multidrug resistant isolates were sub-cultured onto this chromogenic medium, incubated overnight at 35°C and interpreted as directed by the manufacturer.

### Chemical analysis for antimicrobial residues

Upon arrival, the aqueous samples were filtered using vacuum filtration. The samples were poured into a funnel and passed through Whatman 70 mm circular glass microfiber filters. After filtration, the filtrate was transferred into new, clean 1 L amber vials where the pH was adjusted to 2.0 ± 0.5 using 2 M hydrochloric acid and followed by solid phase extraction.

### Solid samples

For each sediment sample, 20 g (wet weight) was placed inside a centrifuge tube and stored in a −20°C freezer prior to freeze drying. The sample was left in the lyophilizer for approximately 48 hours. Once the sediment was completely free of moisture, approximately 1 g (dry weight) was carefully weighed out and transferred to a clean centrifuge tube, to which 20 ml of LCMS grade acetonitrile was added. A blank with no sediment was used as the laboratory control. Next, the sediment and solvent mixtures were sonicated for 30 minutes and then centrifuged for 5 minutes at 3000 rpm. The supernatant was decanted into a clean 250 ml round bottom flask, while the sediment was further mixed with 15 ml of phosphate buffer, pH adjusted to 2.0 ± 0.5 using 2 M hydrochloric acid and additional phosphate buffer, and an additional 20 ml of acetonitrile was added. The mixtures were sonicated and centrifuged again.

The supernatants were decanted and combined with the first supernatants collected in the round bottom flasks. An additional extraction using 15 ml of acetonitrile was performed to each sediment sample with sonication and centrifugation as aforementioned. The combined supernatants were concentrated using a rotary evaporator to reach a final volume of approximately 20 - 30 ml. Finally, 200 ml of reagent water and 500 mg of EDTA was added into the concentrated supernatants which were subjected to solid phase extraction.

### Solid phase extraction

Chromabond HR-X cartridges (Macherey-Nagel Inc. Bethlehem, PA, USA) were conditioned using 5 ml methanol and 5 ml of acidified (pH 3) deionized water at 3 ml/min. The sample extracts were loaded into the solid phase extraction cartridges at 5 mL/min and then washed with 5 ml of acidified deionized water and dried for 30 minutes. The analytes were then eluted using 5 ml of methanol, 5 ml of the 50:50 vol/vol methanol and acetone at 1 ml/min. The extracts were collected in new clean centrifuge tubes and dried under nitrogen. Finally, the samples were resuspended in 200 ml of LCMS grade water with 0.1% formic acid and ammonium acetate with simetone (internal standard at 1 μM). They were sonicated to homogenize and transferred to 2 ml vials for chemical analysis.

### Calibration curves

The internal standard and antibiotics (simetone (internal standard), azithromycin, ciprofloxacin, doxycycline, erythromycin, sulfamethoxazole, tetracycline, and trimethoprim) were re-suspended in Optima grade acetonitrile; diluted to concentrations of 0.001 μM, 0.01 μM, 0.1 μM and 1μM, respectively, with the internal standard at a concentration of 1 μM. Sample peak areas were collected using the Thermo Xcalibur Qual Browser (Thermo Fisher Scientific, Waltham, MA, USA) with selection criteria based on the single reaction monitoring parameters as shown in Table 1. The linear regression line R^2^ values for all standards were above 0.95 generated in Microsoft Excel.

**Table 1:** Mass Spectrometer Parameters Used to Set Up the Single Reaction Monitoring Method on the TSQ Endura^™^

**Standard**	**Retention time (min)**	**Precursor (m/z)**	**Product (m/z)**	**Collision energy**
**Azithromycin**	4.84	749.50	591.20	32.702
**Ciprofloxacin**	4.44	332.20	314.00	21.124
**Doxycycline**	5.51	445.10	428.00	19.050
**Erythromycin**	5.16	734.40	576.22	22.135
**Simetone (internal standard)**	1.50	192.20	174.10	13.388
**Sulfamethoxazole**	4.44	254.10	156.00	16.219
**Tetracycline**	4.33	445.20	410.10	20.466
**Trimethoprim**	4.17	291.20	230.00	24.258

### Ultra-high performance liquid chromatography-mass spectrometry

Ultra-high performance liquid chromatography separation was performed on a Dionex Ultimate 3000 (Thermo Fisher Scientific). The injection volume of water samples and standards was 30 μl loaded onto a pre-equilibrated Luna Omega 1.6 μm Polar C18, 100 Å, 100 × 2.1 mm (Phenomenex, Torrance, CA) with the respective security guard column, at 10% solvent B (60% acetonitrile, 38% methanol, 2% water, 20 mm ammonium acetate, 0.2% formic acid) and 90% solvent A (100% water, 20 mm ammonium acetate, 0.2% formic acid) at a constant flow rate of 0.4 ml/min. The column temperature was set at 55°C throughout the entirety of the run. Injected samples were washed for 1 minute in 10% solvent B before beginning the elution gradient. Solvent B was increased from 10% to 75% over 1 minute, maintained at 75% for 6 minutes, increased to 95% over 1 minute and maintained at 95% solvent B for the remainder of the 10-minute run. To re-equilibrate the column to starting conditions, a blank injection (95% solvent B, 5% solvent A) was inserted between each sample. The blank injection began at 95% solvent B for 1 minute. The gradient decreased to 10% solvent B over 1 minute and remained constant for 3 minutes. Tandem mass spectrometry data were acquired using TSQ Endura^™^ triple quadrupole mass spectrometer (Thermo Fisher Scientific) equipped with a heated electrospray ionization source with the following ionization parameters: positive ion (V): 4300; sheath gas (arb): 10; aux gas (arb): 5; sweep gas (arb): 1; ion transfer tube temperature (°C): 275; vaporizer temperature (°C): 20. Samples and standards were analyzed using single reaction monitoring mode with a retention time window of 3 minutes. Internal standard and antibiotics were optimized for product ion and collision energies. Table 1 lists the mass spectrometer parameters used to set up the single reaction monitoring method on the TSQ Endura^™^. In instances where the mass over charge (m/z) value was the same between two standards, an additional m/z value was applied to confirm detection.

### Statistical analysis

All data were entered into Microsoft Excel. Statistical Package for the Social Sciences software (IBM SPSS Statistics 24^®^) was used to determine descriptive analysis of ESBL genes and carbapenem resistant enterobacteria isolates, and the frequency distribution according to geographical sampling sites and susceptibility patterns among bacterial isolates. For inferential analysis, one sample T-test was selected to compare the mean of all water samples with the standard value of 126/100 ml. Probabilities less than 5% (p < 0.05) were considered statistically significant.

## Results

**Figure 2:**
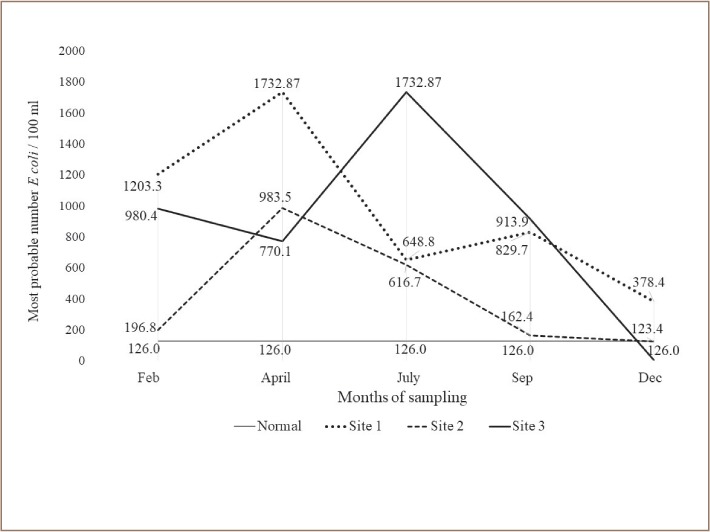
Most probable number (MPN) values of E coli (per 100 ml) identified from water samples collected from the Rio Grande River. Site 1: Sunland park area near waste water treatment plant (NM); Site 2: Riverbend/Courchesne area (Texas); Site 3: Anapra area (Juarez, Mexico and Texas). Water fecal contamination was determined by the IDEXX Colilert MPN method.

Fecal contamination was found to be consistently higher than the standard set by the Texas Commission of Environmental Quality (TCEQ) (126 MPN/100 ml),^37^ except for samples collected from Site 2 during the months of September and December. Fecal contamination appears to increase during irrigation season in the months of April and July, specifically in Site 3, where levels exceed the standard by 13.7 times. For the months of September, December and February the MPN values decreased closer to the TCEQ standard. The mean MPN value (751.5) was higher than the TCEQ set standard of 126 per colony-forming unit/ml for this segment of the Rio Grande River (p<0.01) *(Figure 2)*.

### Extended spectrum β-lactamase encoding genes and mobile genetic elements directly from water samples and bacterial isolates

**Figure 3:**
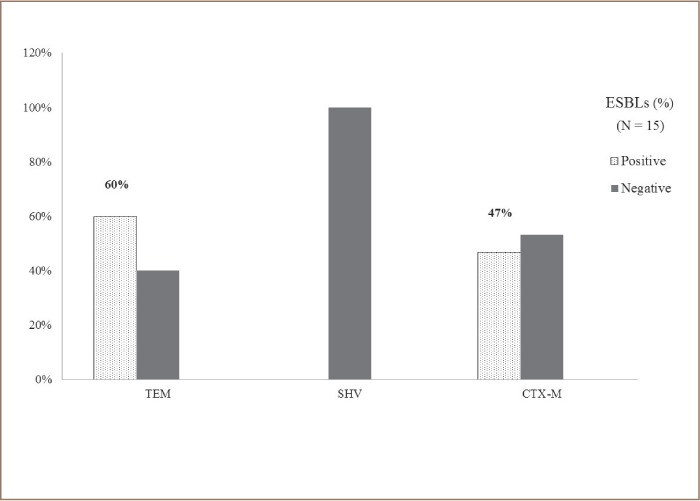
Percentage of ESBL genes (TEM, SHV and CTX-M) identified from water samples (N= 15) from the Rio Grande River. Amplification of selected extended spectrum beta-lactamase (ESBL) genes was accomplished by PCR as described in methods section.

**Figure 4:**
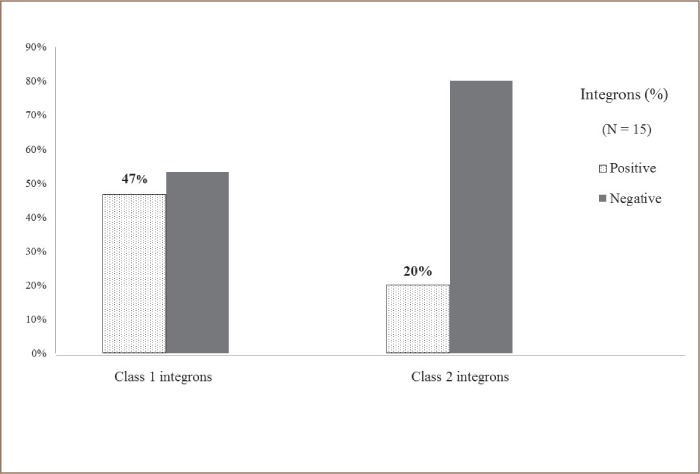
Percentage of Class 1 and Class 2 integrons identified from water samples (N=15) from the Rio Grande River. Amplification of selected Class 1 and 2 integrons was accomplished by PCR as described in the Methods section.

Fifteen water samples were collected from three sites described in the Methods section. Extended spectrum β-lactamase genes were detected in 11 water samples (73.0%). From these, 7 (46.7%) tested positive for the identification of the CTX-M gene, and 9 (60.0%) for the TEM gene. The SHV gene was not identified in any of the water samples that were directly analyzed for the identification of ESBL genes *(Figure 3)*. Class 1 and Class 2 integrons were identified in 11 samples (73.3%). Class 1 integrons were the most prevalent *(Figure 4)*.

A total of 28 bacterial isolates were selected for molecular analysis of ESBL genes. Four (14.3%) carried the SHV gene and 13 (46.4%) the TEM gene. Four isolates (14.3%) carried both SHV and TEM genes and only one isolate (3.6%) carried both TEM and CTX-M genes, as shown in Table 2.

**Table 2:** Frequency Distribution of ESBL Genes (SHV, TEM and CTX-M) Among 28 Multidrug Resistance Isolates

**ESBL gene**	**Frequency**	**(%)**
**TEM**	13	46.4
**Non-ESBL**	6	21.4
**SHV**	4	14.3
**SHV, TEM**	4	14.3
**TEM, CTX-M**	1	3.6
**CTX-M**	0	0.0
**Total**	28	100.0

Abbreviation: ESBL, extended spectrum beta-lactamase

### Identification and antimicrobial susceptibility of bacterial isolates

A total of 310 isolates were collected and processed by the MicroScan autoSCAN-4 system. Genus and species of 142 gram negative and gram positive isolates belonging to 18 bacterial genera were identified with probabilities of correct identification ranging from 92.72–99.99% as shown in Supplemental Material. From these, 91 isolates showed resistance to at least two synergistic antibiotic combinations (amoxicillin/potassium clavulanate, piperacillin/tazobactam, trimethoprim/sulfamethoxazole, ticarcillin/clavulanate and ampicillin/sulbactam). One hundred and one (101) isolates were resistant to at least four individual antibiotics. Eleven of the isolates were identified as ESBL-producing bacteria by MicroScan and 21 of the isolates were identified to be resistant to 20 or more individual antibiotics. Fifteen of these isolates were isolated in Sites 1 and 3. Multidrug resistant isolates were found in all three sites. Fourteen out of the 21 multi-resistant isolates were identified as E. coli or Klebsiella pneumonia. A description of 142 bacterial isolates identified in this study and antibiograms can be found in the Supplemental Material. Extended spectrum β-lactamase-producing organisms and carbapenem-resistant enterobacteria are shown in Table 3.

**Table 3:** Frequency Distribution of Positive ESBLs and Carbapenem-Resistant Enterobacteria Among 28 Multidrug Resistant Isolates

**Microorganism**	**TEM**	**SHV**	**CTX-M**	**Carbapenem-resistant enterobacteria**	**Total**
**Aeromonas hydrophilia**	1	-	-	-	1
**Cedecea davisae**	1	-	-	-	1
**Citrobacter freundii*c.***	1	-	-	1	1
**Escherichia coli**	8	1	-	-	11
**Klebsiella pneumoniae**	4	7	-	8	9
**Klebsiella oxytoca**	2	-	1	2	2
**Leminorella*sp.***	-	-	-	-	1
**Vibrio fluvialis**	1	-	-	-	1
**Vibrio parahaemolyticus**	-	-	-	-	1
**Total**	18	8	1	11	28

Abbreviation: ESBL, extended spectrum beta-lactamase

### Chemical analysis for antibiotics

**Figure 5:**
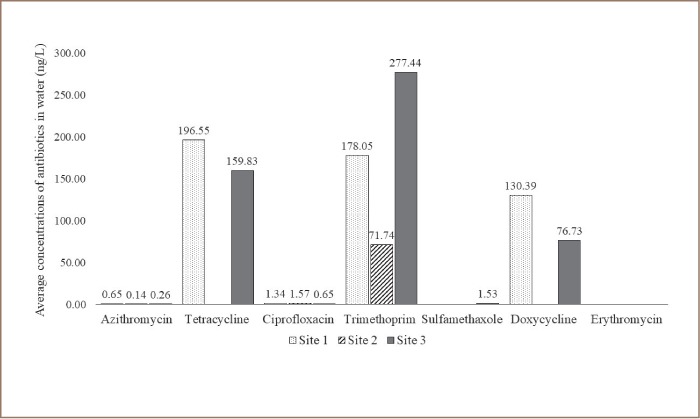
Average concentrations (ng/L) of antimicrobial residues identified from water samples (N= 15) from the Rio Grande River. Antibiotic residues were determined by liquid chromatography and mass spectrometry.

**Figure 6:**
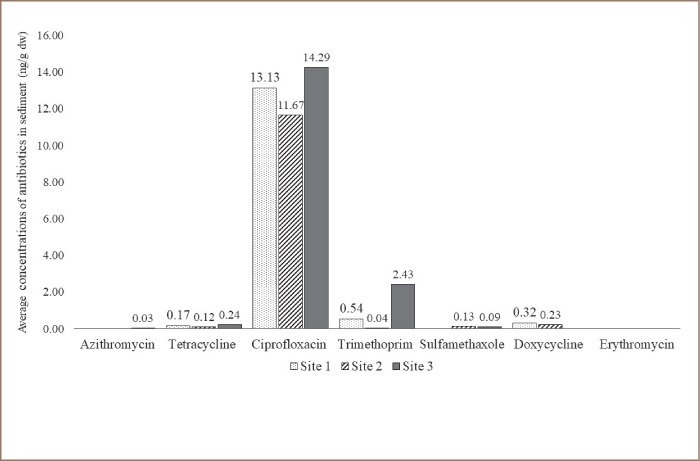
Average concentrations of antimicrobial residues (ng/g) identified from sediment samples (N= 15) from the Rio Grande River. Antibiotic residues were determined by liquid chromatography and mass spectrometry.

The analytical method implemented was able to detect azithromycin, ciprofloxacin, doxycycline, erythromycin, sulfamethoxazole, tetracycline, and trimethoprim. The limit of detection was 0.001 μM for all antibiotics. Antibiotics were present in water and sediment in the range of 0.38 ng/L - 742.73 ng/L and 0.39 ng/l - 66.3 ng/g dry weight, respectively. From the seven antibiotics analyzed, ciprofloxacin was the most commonly detected in sediment and trimethoprim was most frequently detected in water. As shown in Table 4 and 5, at least one antibiotic was found in 80% and 100% of all sediment and water samples, respectively. Samples collected in February usually had the highest level of antibiotics and samples collected in September had the least amount of antibiotics. Site 3 had the highest levels of antibiotics found in water and sediment *(Figures 5 and 6)*. Antibiotics were found in 92% of both water and sediment samples. Site 3, in general, had the highest levels of antibiotics found in both water and sediment and the most commonly found antibiotics in water and sediment combined were trimethoprim, ciprofloxacin, doxycycline, with 63%, 58%, and 21% occurrence, respectively.

## Discussion

The present pilot study identified and characterized MDR bacteria, ESBL genes (TEM, CTX-M, SHV), Class 1 and 2 integrons, and measured antimicrobial residues present in waters of the Rio Grande River along El Paso, TX, Sunland Park, NM and Juarez, Mexico, which borders this area.

Water quality in the Rio Grande varies significantly along the border region throughout the year. Fecal coliforms and E. coli values ranged between 123 to 1732.87 colony-forming unit/ml. The TCEQ standard for E. coli is set at 126 colony-forming unit/ml.^37^ The E. coli counts observed during the 12-month water sample collection period changed greatly *(Figure 2)*. All water samples collected were above the standard set by TCEQ, except during the month of December, possibly due to climate change conditions such as lower temperatures and/or water flow. The highest E. coli numbers occurred during the months of February, April, July and September. Site 1 and Site 2 exceeded the limit by 13.7 and 7.8 times, respectively, for the month of April. Site 3 exceed the standard limit by 13.7 times during the month of July. The lowest E. coli/coliform bacteria numbers were found in the month of December for all of the sites. These results are in agreement with recent reports from the National Park Service, which finds that the water quality in the Rio Grande River is highly variable and high numbers of bacteria occur during the months of June through October due to runoff, animal waste and other pollutants during the monsoon season.^38^ Interestingly, in contrast to previous studies conducted in one of our laboratories, the highest number of coliform bacteria occurred during the months of November and December.^26^,^39^ This significant difference shows the great variability of water flow in the Rio Grande River as influenced by climatological conditions, which dictates irrigation seasons.^40^ An additional factor influencing fecal contamination could be that the adherence to regulations on water quality standards differs significantly in the sampled area, which includes three states (New Mexico, Texas, Chihuahua) and two countries (US and Mexico), impacting coliform levels. Water pollution awareness must be disseminated to people who use the river for recreational purposes as they may be exposed to MDR bacteria, leading to changes in their microbiota and they may become chronic carriers of these AR enterobacteria. Additional public health concerns may include a higher risk of gastrointestinal infections and possible lower response to antimicrobial therapy.

The identification of 142 isolates with 92–99.99% probability of correct identification, with the majority being highly resistant to multiple individual and synergistic antibiotic combinations used to treat serious infections, confirmed the potential health hazards of this segment of the river. The high number of enteric and other bacteria found is probably due to the effluents of wastewater treatment plants near the sampled area, as well as animal and human waste that impact the river. As previously reported, increased anthropogenic activities are contributors to AR bacteria, and water treatment plants are considered hotspots of AR.^10^,^41^–^43^ This segment of the Rio Grande River is also impacted by migratory birds and Sites 1 and 2 are surrounded by agricultural fields, dairy farms and horse breeding facilities *(Figure 1)*. Of greatest concern is Site 3, where children, mainly from the Mexican side, use this segment of the river for recreational activities.

The presence of plasmid-mediated β-lactamase and cephalosporin resistance was studied by the amplification of TEM, CTX and SHV genes by multiplex polymerase chain reaction.^36^ The results in the present study showed that the percentage of TEM (60%) and CTX-M (40%) correlate well with other reports from urban rivers around the world and areas where poor sanitation is a problem.^44^–^47^ In agreement with other studies, the SHV gene was only detected in Klebsiella species.^28^,^44^

Class 1 and 2 integrons have been reported as a proxy of environmental pollution.^41^,^48^,^49^ In the present study, integrons were analyzed directly from water samples. Class 1 integron was the most prevalent (46.7%). Together, Class 1 and 2 integrons accounted for 73% of water samples collected. Previous chemical analyses in the Rio Grande River within the New Mexico-Texas region showed a high salinity and boron content.^50^,^51^ Both boron and salinity are described as markers of wastewater treatment plant effluents and correlate well with the presence of Class 1 integrons.^17^

Antibiotics measured along the 26 km selected area of the Rio Grande were present in water and sediment in the range of 0.38 ng/L - 742.73 ng/L and 0.39 ng/l - 66.3 ng/g dry weight, respectively. From the seven antibiotics analyzed, ciprofloxacin was the most commonly detected in sediment and trimethoprim was most frequently detected in water. The levels of antibiotics detected for each site match the number of MDR bacteria-isolated antibiotic profiles during the months of April through September. Furthermore, the highest levels of antibiotics were found in Site 3, which is a hotspot for potential gastrointestinal and opportunistic infections and will likely affect surrounding populations that use the river for recreational activities. Consistent with our study, reports from antibiotic-contaminated water worldwide indicate that levels of trimethoprim, sulfamethoxazole followed by ciprofloxacin are high, and concentrations of ciprofloxacin have been reported to be as high as 6.5 mg/L.^52^,^53^ Since antibiotics are not completely degraded after wastewater treatment, effluents that reach the river along with agrochemicals and animal waste contribute to antibiotic contamination. Moreover, the low levels of antibiotics plus the presence of heavy metals will impact bacterial communities, exerting selection pressure to maintain plasmids and drug resistance.^27^,^28^,^54^–^57^

Our findings suggest that the Rio Grande River is a hotspot for the presence of ARG, mobile genetic elements and antimicrobial residues. As water from the Rio Grande River is the main source of potable water and a site of recreation for some of the population, this area may represent a public health concern. Another important aspect of the border area is heavy traffic. The US-Mexico border, specifically between El Paso-Ciudad Juarez is one of the busiest international crossings in the world. In 2017 alone, the US Department of Transportation reported a total of 41 million crossings, excluding those related to commercial purposes.^58^ Metagenomic studies have shown that travel is an important factor that contributes to the rapid dissemination of AR among continents, as the human gut microbiome functions as a carrier and transport of MDR bacteria.^59^,^60^

Currently, the health risks associated with continuous exposure to antimicrobial residues and ARG from water sources and through environmental contact is largely unknown and a very important topic of research. The challenge to health risk assessment and management is the lack of standardization and the need to establish baselines for minimal health risk exposure. More data is needed to investigate the fate and effects of antimicrobials in the environment.

## Conclusions

The 26 km segment of the Rio Grande River from Sunland Park, NM to El Paso, TX and Juarez, Mexico is an area of concern due to poor water quality. The presence of multi-drug resistant bacteria, antibiotics and mobile genetic elements may be a health hazard for the surrounding populations of this binational border region. Further research is needed to critically evaluate antimicrobial gene transfer and evaluate the health risk in these border populations. Policies need to be developed for appropriate management of the environmental natural resources in this border region.
