# Supplementary material for: Assessment of Antibiotic Levels, Multi-Drug Resistant Bacteria and Genetic Biomarkers in the Waters of the Rio Grande River Between the United States-Mexico Border
Source: J Health Pollut. 2019 Aug 22;9(23):190912. doi: 10.5696/2156-9614-9.23.190912 (PMC6711330; doi:10.5696/2156-9614-9.23.190912)
Supplement: Supplementary file 1 [file Fuentes_Supplemental_Material.docx]

| Supplemental Material  Table 1: Identification and Antibiotic Resistance of Bacterial Isolates | | | | | | | | | | | | | | | |  |  |  |
| --- | --- | --- | --- | --- | --- | --- | --- | --- | --- | --- | --- | --- | --- | --- | --- | --- | --- | --- |
|  | **Isolate identification/**  **probability of correct identification** | **Number of Isolates** | | | | | | | | | | | | |  | | | |
| Month  February |  | **Total** | | | | **Resistant to two or more synergistic combinations** | | | **Resistant to 4 or more individual antibiotics** | | | **Resistant to 20 or more individual antibiotics** | | | **ESBL** | | | |
|  | *Enterococcus durans/hirae*/99.99% | 6 | | | 0 | | | 3 | | | 0 | | | 0 | | | |  |
|  | *Aeromonas hydrophila* complex/99.99% | 8 | | | 1 | | | 0 | | | 0 | | | 0 | | | |  |
|  | *Yersinia enterocolitica* group/99.99% | 1 | | | 0 | | | 1 | | | 0 | | | 0 | | | |  |
|  | *Escherichia coli*/99.99% | 1 | | | 0 | | | 0 | | | 0 | | | 0 | | | |  |
|  | *Vibrio fluvialis*/99.99% | 1 | | | 1 | | | 1 | | | 0 | | | 0 | | | |  |
| April | *Vibrio fluvialis/*97.58-99.99% | 3 | | | 3 | | | 1 | | | 0 | | | 0 | | | |  |
|  | *Vibrio parahaemolyticus*/99.87% | 1 | | | 1 | | | 1 | | | 0 | | | 0 | | | |  |
|  | *Aeromonas hydrophila* complex/98.44-99.99% | 9 | | | 3 | | | 1 | | | 0 | | | 0 | | | |  |
|  | *Escherichia coli*/99.99% | 5 | | | 3 | | | 3 | | | 3 | | | 1 | | | |  |
|  | *Pseudomonas fluorescens*/putida/99.99% | 1 | | | 0 | | | 1 | | | 0 | | | 0 | | | |  |
|  | *Pseudomonas aeruginosa*/99.99% | 1 | | | 1 | | | 1 | | | 0 | | | 0 | | | |  |
|  | *Cedecea davisae*/99.99% | 1 | | | 1 | | | 1 | | | 1 | | | 0 | | | |  |
|  | *Leminorella species*/99.99% | 2 | | | 2 | | | 2 | | | 2 | | | 0 | | | |  |
|  | *Achromobacter xylosoxidans*/99.99% | 1 | | | 1 | | | 1 | | | 0 | | | 0 | | | |  |
|  | *Enterococcus faecium*/99.50% | 1 | | | 0 | | | 1 | | | 0 | | | 0 | | | |  |
|  | *Staphylococcus cohnii* subsp. Cohnii/99.99% | 1 | | | 1 | | | 1 | | | 0 | | | 0 | | | |  |
|  | *Staphylococcus epidermidis*/99.99% | 1 | | | 0 | | | 0 | | | 0 | | | 0 | | | |  |
| July | *Escherichia coli*/94.4-99.99% | 8 | | | 5 | | | 5 | | | 5 | | | 5 | | | |  |
|  | *Klebsiella pneumonia*/99.99% | 4 | | | 4 | | | 4 | | | 4 | | | 4 | | | |  |
|  | *Aeromonas hydrophila* complex/99.99% | 1 | | | 1 | | | 1 | | | 0 | | | 0 | | | |  |
|  | *Cedecea davisae*/99.99% | 1 | | | 1 | | | 1 | | | 1 | | | 0 | | | |  |
|  | *Staphylococcus xylosus*/99.99% | 4 | | | 4 | | | 4 | | | 0 | | | 0 | | | |  |
| September | *Klebsiella pneumoniae*/99.99% | 5 | | | 0 | | | 0 | | | 0 | | | 0 | | | |  |
|  | *Enterobacter cloacae*/99.99% | 3 | | | 3 | | | 3 | | | 0 | | | 0 | | | |  |
|  | *Escherichia coli/*99.99% | 2 | | | 0 | | | 0 | | | 0 | | | 0 | | | |  |
|  | *Staphylococcus sciuri*/94.42-99.99% | 20 | | | 19 | | | 20 | | | 2 | | | 0 | | | |  |
|  | *Staphylococcus auricularis*/95.57% | 1 | | | 0 | | | 0 | | | 0 | | | 0 | | | |  |
|  | *Staphylococcus xylosus*/99.99% | 2 | | | 2 | | | 2 | | | 0 | | | 0 | | | |  |
|  | *Staphylococcus aureus*/95.24-98.99% | 2 | | | 0 | | | 1 | | | 0 | | | 0 | | | |  |
|  | *Staphylococcus hominis* subsp. Hominis/99.99% | 1 | | | 1 | | | 1 | | | 0 | | | 0 | | | |  |
|  | *Staphylococcus schleiferi* subspecies schleiferi/97.76% | 1 | | | 1 | | | 1 | | | 0 | | | 0 | | | |  |
| December | *Klebsiella* *pneumoniae*/99.99% | 2 | | | 2 | | | 2 | | | 2 | | | 1 | | | |  |
|  | *Enterobacter cloacae*/99.99% | 1 | | | 1 | | | 1 | | | 1 | | | 0 | | | |  |
|  | *Pseudomonas luteola*/99.99% | 1 | | | 1 | | | 1 | | | 0 | | | 0 | | | |  |
|  | *Vibrio metschnikovii*/99.99% | 1 | | | 1 | | | 1 | | | 0 | | | 0 | | | |  |
|  | *Vibrio cholerae*/99.99% | 1 | | | 0 | | | 0 | | | 0 | | | 0 | | | |  |
|  | *Cedecea neteri*/99.77% | 1 | | | 0 | | | 1 | | | 0 | | | 0 | | | |  |
|  | *Burkholderia cepacia* Complex/99.99% | 1 | | | 0 | | | 0 | | | 0 | | | 0 | | | |  |
|  | *Sphingobacterium multivorum*/99.99% | 2 | | | 2 | | | 2 | | | 0 | | | 0 | | | |  |
|  | *Stenotrophomonas maltophilia*/99.99% | 1 | | | 0 | | | 0 | | | 0 | | | 0 | | | |  |
|  | *Chryseobacterium indologenes*/99.99% | 1 | | | 1 | | | 1 | | | 0 | | | 0 | | | |  |
|  | *Acinetobacter baumannii* complex/*haemolyticus*/99.78% | 1 | | | 1 | | | 1 | | | 0 | | | 0 | | | |  |
|  | *Sphingomonas paucimobilis*/99.99%% | 1 | | | 1 | | | 1 | | | 0 | | | 0 | | | |  |
|  | *Staphylococcus xylosus*/99.99% | 7 | | | 7 | | | 7 | | | 0 | | | 0 | | | |  |
|  | *Staphylococcus sciuri*/99.27-99.99% | 5 | | | 5 | | | 5 | | | 0 | | | 0 | | | |  |
|  | *Staphylococcus aureus*/98.99-99.99% | 2 | | | 0 | | | 2 | | | 0 | | | 0 | | | |  |
|  | *Staphylococcus epidermidis*/99.99% | 4 | | | 2 | | | 4 | | | 0 | | | 0 | | | |  |
|  | *S. auricularis*/92.76-99.16% | 2 | | | 2 | | | 2 | | | 0 | | | 0 | | | |  |
|  | *S. haemolyticus*/99.99% | 3 | | | 2 | | | 3 | | | 0 | | | 0 | | | |  |
|  | *Staphylococcus schleiferi subspecies schleiferi*/99.99% | 3 | | | 2 | | | 3 | | | 0 | | | 0 | | | |  |
|  | *S. schleif*-coag/99.99% | 1 | | | 0 | | | 0 | | | 0 | | | 0 | | | |  |
|  | *S. hominis*-novo/99.00% | 1 | | | 1 | | | 1 | | | 0 | | | 0 | | | |  |
|  | *Staphylococcus hyicus*/99.99% | 1 | | | 1 | | | 1 | | | 0 | | | 0 | | | |  |
| TOTAL |  | | 142 | 91 | | | 101 | | | 21 | | | 11 | | | |  |  |

Abbreviation: ESBL, extended spectrum beta-lactamase
